# Supplementary material for: Multi-structure Cortical States Deduced From Intracellular Representations of Fixed Tactile Input Patterns
Source: Front Cell Neurosci. 2021 Jun 14;15:677568. doi: 10.3389/fncel.2021.677568 (PMC8236821; doi:10.3389/fncel.2021.677568)
Supplement: SUPPLEMENTARY FIGURE 1 — Stability of identified clusters across the full range of parameter settings. (A) Mean agreement between surrounding elements. The position of each center element was defined by the values of its “Threshold” and “Overshoot” parameters. The color code indicates the average number/proportion of response members of the center element that fell into the same clusters as in each of the eight surrounding elements, across all clusters identified. Data for Neuron#5, pattern S5 (same as in Figures 2, 3). (B) Maximal agreement between surrounding elements. Same analysis as in (B), but instead the highest level of agreement between two clusters is illustrated. The color code here instead indicates the average max agreement between the central element and its eight surrounding elements. [file Data_Sheet_1.PDF]

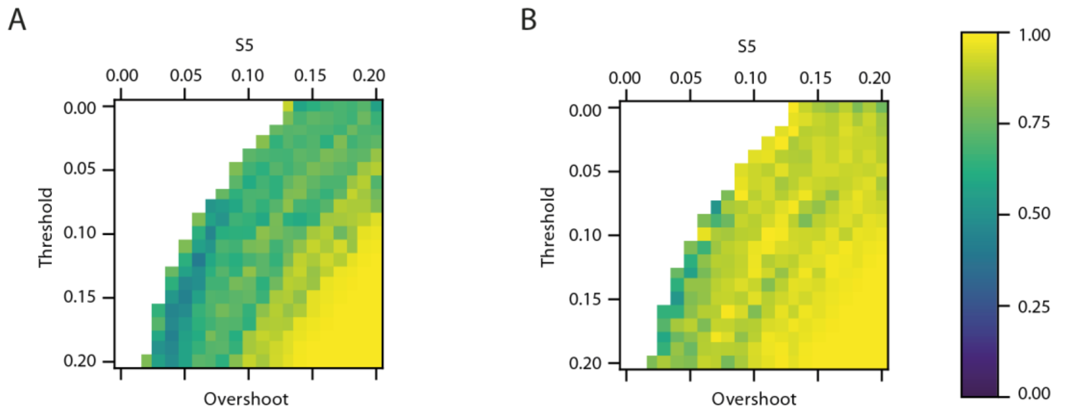

Supplementary Figure 1. Stability of identified clusters across the full range of parameter settings. (**A**) Mean agreement between surrounding elements. The position of each center element was defined by the values of its 'Threshold' and 'Overshoot' parameters. The color code indicates the average number/proportion of response members of the center element that fell into the same clusters as in each of the eight surrounding elements, across all clusters identified. Data for Neuron#5, pattern S5 (same as in Figs 2 and 3). (**B**) Maximal agreement between surrounding elements. Same analysis as in B, but instead the highest level of agreement between two clusters is illustrated. The color code here instead indicates the average max agreement between the central element and its eight surrounding elements.
